# Mental Health Literacy, Positive Mental Health, and Help-Seeking Intention Among Undergraduate Nursing Students: A Correlational Study

**DOI:** 10.3390/nursrep16100369

**Published:** 2026-10-19

**Authors:** Luís Loureiro, Ana Teresa Pedreiro

**Affiliations:** 1 Health Sciences Research Unit: Nursing (UICISA E), Nursing School of the University of Coimbra (ESEUC), University of Coimbra, 3004-011 Coimbra, Portugal; 2 School of Economics, Management and Political Science, University of Minho, 4710-057 Braga, Portugal; teresa.pedreiro@eeg.uminho.pt

**Keywords:** mental health literacy, positive mental health, help-seeking intention, nursing, youth

## Abstract

**Background/Objectives:** Mental health literacy (MHL) is considered an important resource for promoting mental health, facilitating the recognition of mental health problems, reducing stigma, and encouraging appropriate help-seeking behaviours. Among undergraduate nursing students, MHL may also be associated with positive mental health and a greater willingness to seek professional support when needed. This study aimed to examine the associations between mental health literacy, positive mental health, and help-seeking intention among undergraduate nursing students. **Methods:** A correlational study was conducted with 388 nursing students (50.3% enrolled in the second year and 49.7% enrolled in the fourth year), predominantly women (84.0%), with a mean age of 21.76 years (SD = 5.00) and a median of 21.0 years. **Results:** Nursing students showed relatively high levels of mental health literacy, positive mental health, and help-seeking intention. Small but significant multivariate main effects of year of study and familiarity with mental illness, without interaction, were found on the combined set of global scores. In univariate follow-up analyses that remained significant after Bonferroni correction, students with high familiarity (both close contact with and personal experience of mental health problems) showed lower positive mental health, and fourth-year students reported higher help-seeking intention than second-year students; small differences in mental health literacy and mental health–promoting knowledge did not survive correction. Canonical correlation analysis identified a single significant, modest association between mental health literacy, mainly its attitudinal component and mental health–promoting knowledge, and positive mental health (Rc = 0.42; redundancy indices of 3.5% and 5.8%). In the hierarchical regression predicting help-seeking intention, knowledge of risk factors and causes and positive mental health dimensions (problem-solving and personal fulfilment, interpersonal relationship skills) were positive predictors, as were attitudes that promote recognition. Overall, the final model explained a modest proportion of the variance (R^2^ = 0.162; adjusted R^2^ = 0.126). **Conclusions:** Nursing students display an overall favourable profile of mental health literacy, positive mental health, and help-seeking intention, already high among second-year students; self-selection into the profession is one possible explanation that requires longitudinal confirmation. Differences by year of study and familiarity were small: fourth-year students reported higher help-seeking intention, and students with high familiarity reported lower positive mental health.

## 1. Introduction

The mental health of higher education students has become a growing concern for higher education institutions. The available evidence, derived from recent systematic reviews and meta-analyses, documents high rates of psychological distress, anxiety, and depressive symptoms in this population, alongside a gap between healthcare needs and the proportion of students seeking specialised support [1].

This gap between perceived need and service utilisation has been interpreted not only as a problem of accessibility, often associated with economic and financial factors, but also as a problem related to limited health literacy, associated with stigmatising attitudes towards mental health problems. In other words, this is a psychosocial phenomenon that precedes and, to some extent, conditions help-seeking behaviour in mental health.

In Portugal, these issues led to the establishment of the Programme for the Promotion of Mental Health in Higher Education, including the Psychologist Voucher Programme, aimed at providing psychological support to higher education students in Portugal.

Among nursing students, this vulnerability has particular characteristics arising from the nature of their programme. Nursing education combines demanding academic requirements with clinical training that exposes students at an early stage to vulnerability, suffering, serious illness, and patient deaths, at a stage when they do not yet have the professional experience or emotional regulation strategies that characterise experienced nurses.

Studies conducted in different European contexts show higher levels of perceived stress and psychopathological symptoms than those observed among students enrolled in other degree programmes. This difference stems from factors such as workload, assessment in clinical settings, fear of making mistakes, and difficulties balancing academic and personal life [2]. At the same time, academic distress and depressive symptoms among these students have been found to be associated with personal resources, such as resilience and well-being [3], particularly insofar as the way students cope with stress is associated with their level of positive mental health [4].

A further element is the cultural dimension of professional socialisation in nursing, or the process of becoming a nurse. The ethos of the profession values, in addition to technical competencies, skills such as empathic communication and responsiveness to others, as well as self-sufficiency and resilience. Although these attributes are desirable, they may become an obstacle when students are confronted with their own psychological distress.

Recognising the need for help may be experienced as incompatible with their developing professional identity, resulting in increased self-stigma, which may, for example, lead to the concealment of signs and the silencing of symptoms.

A recent study conducted with Portuguese nursing students during periods of academic transition identified this pattern. The study found moderate levels of depression literacy, persistent stigmatising beliefs, and perceived barriers to mental health help-seeking, particularly a preference for self-reliance and fear of disclosure among peers and teaching staff were particularly prominent [5]. These findings are consistent with the international literature on stigma among nursing students, which describes negative attitudes towards mental illnesses and their association with lower willingness to seek specialised mental health support [6,7]. These challenges are further compounded both by potential difficulties in managing mental health issues throughout their educational pathway and by the fact that, in the future, these students will work on the frontline of healthcare delivery and may therefore contribute to the early detection of mental health problems and appropriate referral. Thus, their mental health literacy, attitudes, and personal experiences of help-seeking may influence the quality of the responses they will provide in the future to healthcare users [8].

### From Mental Health Literacy to Self-Care and Help-Seeking

The concept of MHL was introduced by Jorm and colleagues [9] to describe a set of beliefs and knowledge about mental disorders that enables their recognition, management, and/or prevention [9]. The concept now encompasses five components, namely: (a) knowledge of when a person is developing a mental disorder; (b) knowledge of how to prevent mental health problems; (c) knowledge of help-seeking options and available treatments; (d) knowledge of the effectiveness of self-help strategies for moderate problems; and (e) skills to provide first aid to other people who are developing a mental disorder or experiencing a crisis related to their mental health [10].

More recently, particularly through the work of Kutcher and colleagues [11,12], the concept of MHL has been further refined.

Building on the concept of health literacy and the pioneering proposal by Jorm and colleagues [9], these authors [11,12] added the term “positive” to MHL and defined it as an individual’s awareness of how they can achieve and maintain good mental health [13], reflected in the knowledge and skills that support mental health promotion.

Accordingly, PMHL encompasses the following components: (a) understanding how positive mental health can be achieved and maintained; (b) understanding mental disorders and their treatments; (c) reducing stigma associated with mental illnesses; and (d) improving the effectiveness of help-seeking (knowing when and where to seek help and developing skills aimed at improving mental healthcare and management abilities) [12].

The roots of positive mental health can be traced to the pioneering work of Jahoda [14], and more recently to Keyes’ dual-continuum model [15]. While Jahoda provides the conceptual foundation for positive mental health, Keyes formalised and empirically validated the structure of well-being and its relative independence from psychopathology. What connects these approaches is their rejection of the unidimensional model that views mental health merely as the absence of mental illness.

The scope of this conceptualisation of mental health and well-being is reflected in the WHO’s 2022 World Mental Health Report, entitled “Transforming mental health for all” [16]. Mental health becomes an inseparable element of human experience, underlying our thoughts, emotions, and behaviours. Far from being merely a clinical concern, it is asserted as a fundamental right and a strategic pillar for the development of individuals and groups. This subjective dimension is as vital as physical integrity, operating continuously and underpinning our ability to make decisions and shape the environment in which we live. Mental health can therefore be understood as a dynamic state of balance and capabilities that enables individuals to live, relate to others, and face challenges; well-being describes how positive and satisfactory a person’s subjective experience and psychological functioning are. For assessment and intervention, many authors recommend measuring and promoting both: reducing distress/disorder while simultaneously increasing well-being [16].

In the field of nursing, the work revisiting positive mental health has been carried out by Teresa Lluch [17,18]. The author defines positive mental health through four fundamental pillars: positive mental health is a dynamic and fluctuating construct; the coexistence of positive and negative aspects (whether emotional, cognitive, or behavioural); the identification of warning signs requiring monitoring; and systemic organisation, in which different factors interact to compensate for potential internal imbalances within the model.

The operationalisation of this model draws on the individual’s capacity to adapt, and is divided into two main spheres:

(a) Individual level: Priority is given to the perception of satisfaction, emotional management, independence, and the search for effective solutions in the face of adversity and challenges;
(b) Interpersonal level: This is based on the quality of social interactions and the propensity for supportive and cooperative behaviours among individuals.

A strength of Lluch’s approach [19] lies in proposing a feasible form of mental health, which emphasises maintaining autonomy and self-actualisation within the limitations of each context. Positive mental health is therefore the ability to manage relationships and emotions functionally, even in the face of adverse circumstances [20].

The relationship between MHL and positive mental health is not linear, as some of the literature might suggest, even though the term positive mental health literacy is used to refer to knowledge and skills that enable individuals to promote and maintain their own mental health. This interpretation supports the idea that positive mental health and MHL are not independent domains, but rather partially overlapping dimensions of the same competence, namely knowing how to care for oneself [21].

With regard to mental health help-seeking intention (MHHI), this variable has been studied in terms of intention rather than actual behaviour. According to the Theory of Planned Behaviour, intention constitutes a proximal determinant of behaviour and is, in turn, determined by attitudes, subjective norms, and perceived behavioural control [22]. The application and validity of this model in the health field have been documented [23], including among nursing students [24].

Among nursing students, the specific characteristics of the degree programme and educational process distinguish them from what is considered the general pattern. As demonstrated by Monton [25], despite being familiar with services and how they operate, students fear the consequences of disclosure, believing that revealing their psychological functioning may be perceived as a vulnerability that renders them incapable of caring for others.

The relationship among MHL, positive mental health, and HSI, has been interpreted in various ways.

MHL is associated with HLI, although the strength of this relationship is modest, limiting claims that higher MHL is associated with seeking and mobilising professional support. The meta-analysis by Özparlak and colleagues [26] identified a moderate positive association between literacy and attitudes towards help-seeking (r = 0.48), but a weaker association with HSI (r = 0.23). These findings suggest that recognising mental health problems and valuing professional help does not necessarily translate into an equivalent willingness to access specialised services.

Nevertheless, a substantial body of studies among higher education students, including nursing students, supports a positive relationship between these variables [27,28,29,30,31,32].

The TPB helps to understand these differences by considering that intention depends on attitudes, subjective norms, and perceived behavioural control [22]. The review by Adams and colleagues reinforces the relevance of these factors, particularly attitudes and perceived control, in explaining help-seeking intention [33].

The relationship between MHL and positive mental health shows a distinct pattern. In the same meta-analysis [26], the association between MHL and mental well-being was small and not statistically significant (r = 0.06, 95% CI [−0,05: 0,16]), suggesting that higher levels of literacy do not necessarily correspond to greater well-being. Consistently, Gorczynski and colleagues [34] observed, among British university students, a positive association between MHL and HSI, but not between MHL and WB. Evidence in the literature indicates a statistically significant relationship, although its strength is modest [35].

Considering these dimensions jointly makes it possible to examine how knowledge about mental health, the experience of well-being, and willingness to seek support in situations of vulnerability are related, without assuming that they develop in parallel or that improvement in one necessarily produces changes in the others, in line with the available evidence. Understanding how MHL, PMH, and help-seeking intention interact among nursing students is considered a necessary step towards informing educational interventions that do not merely convey knowledge about psychopathology, but simultaneously promote well-being and an actual willingness to seek help. Against this background, the objectives of this study were:

(a) To assess MHL, positive mental health, and mental health help-seeking intention, based on a sample of second- and fourth-year nursing students attending a university in the central region of mainland Portugal.
(b) To analyse differences in MHL, PMH, and mental health HSI according to year of study and familiarity with mental illness.
(c) To analyse the relationship between the different dimensions of MHL, including knowledge of mental health and knowledge of mental health promotion, and positive mental health.
(d) To analyse the extent to which year of study, familiarity with mental illness, MHL, and PMH predict mental health HSI.

## 2. Materials and Methods

### 2.1. Research Design

This study employed a quantitative correlational design. The study is reported in accordance with the Strengthening the Reporting of Observational Studies in Epidemiology (STROBE) guidelines for cross-sectional studies.

### 2.2. Sample and Setting

Data were collected at a university located in the central region of mainland Portugal between September and December 2025. All eligible second-year (n = 341) and fourth-year (n = 330) students (N = 671) were invited to participate voluntarily in the study. All fourth-year students had received both theoretical and practical training in psychiatry during the previous year (third year). Second-year students were surveyed at the beginning of the academic year (September–December 2025), shortly after completing the first year of the programme, and had therefore not yet received formal training in psychiatry and mental health.

Of the 671 eligible students, 414 agreed to participate and returned the questionnaire (overall participation rate of 61.70%): 212 second-year students (62.17%) and 202 fourth-year students (61.21%). The remaining 257 students (38.30%) declined to participate or did not respond (129 second-year students, 37.83%; 128 fourth-year students, 38.79%). Of the 414 respondents, 26 (6.28%) were excluded because they did not complete the questionnaire (17 second-year and 9 fourth-year students). The final sample therefore comprised 388 students (195 second-year and 193 fourth-year students), corresponding to a final valid response rate of 57.82% of eligible students (57.18% in the second year and 58.48% in the fourth year). Inclusion criteria were being enrolled in the second or fourth year of the undergraduate nursing programme and providing informed consent; questionnaires with incomplete responses were excluded (complete-case approach). In the final sample, there were no missing data on the study variables, except for one missing value for age.

### 2.3. Data Collection

Data were collected in classrooms using Google Forms. The researchers attended all administration sessions solely to introduce the study, provide access to the questionnaire, and answer any questions.

Access to the questionnaire was provided via a QR code during the academic term. Before completing the questionnaire, students were informed about the study objectives, the voluntary nature of participation, their right to withdraw at any time without any penalty, and the conditions regarding data anonymity and confidentiality. The mean time required to complete the questionnaire was 19.55 min.

### 2.4. Instruments

Sociodemographic questionnaire: This questionnaire included variables such as year of study, age, gender, parents’ education level, and familiarity with mental illness. Familiarity with mental illness was assessed with two dichotomous items (yes/no): close contact, i.e., having a family member or close friend who had experienced mental health problems, and personal experience of a mental health problem.

Mental Health Literacy Scale—MHLS [36]

The MHL assessment scale, in the version translated and adapted to the Portuguese context [37], comprises six components:

- Ability to recognise specific mental health disorders, comprising items 1–8, assesses students’ ability to identify signs, symptoms, and characteristics associated with different mental disorders, enabling the recognition of situations of psychological distress and different types of mental illness (internal consistency measured by the omega coefficient = 0.85).
- Knowledge of ways to seek information about mental health, comprising items 16–19, assesses to knowledge of ways to access mental health information, including the ability to identify credible sources and available resources for obtaining support and clarification (internal consistency measured by the omega coefficient = 0.91).
- Knowledge of causes and risk factors, comprising items 9 and 10, assesses understanding of biological, psychological, social, familial, and environmental factors that may contribute to the development of mental health problems (internal consistency measured by the omega coefficient = 0.78).
- Knowledge of self-help strategies, comprising items 14 and 15, assesses students’ knowledge of self-care strategies, emotional management, and behaviours that promote mental health and positive well-being (PWB) (internal consistency measured by the omega coefficient = 0.86).
- Knowledge of available professional help, comprising items 11–13, assesses knowledge of the different mental health professionals and services, as well as the forms of support and intervention available (internal consistency measured by the omega coefficient = 0.90).
- Attitudes that promote recognition and appropriate self-help, comprising items 20–35, assess attitudes, beliefs, and dispositions favourable to the recognition of mental health problems and the adoption of appropriate help-seeking and self-care behaviours (internal consistency measured by the omega coefficient = 0.86). Items 20 to 28 are reverse-scored, so that higher scores reflect more favourable attitudes [36].

Mental Health Knowledge Schedule—MHKS [38]

The MHKS is a unidimensional measure designed to assess knowledge about mental health, comprising 12 items divided into items related to stigma and items concerning specific mental disorders. Response options range from 1 (Strongly agree) to 5 (Strongly disagree), with an additional option of 6 (Do not know). In this study, the version validated for European Portuguese was used [39]. Internal consistency, assessed using McDonald’s omega, was 0.76.

Mental Health Promoting Knowledge Scale—MHPK-10 [40]

The MHPK-10 assesses individuals’ knowledge of the factors that promote good mental health. It comprises 10 statements rated for accuracy, based on what individuals consider important for maintaining good mental health. Each item has six response options, including “Do not know”, equivalent to zero, and a five-point Likert scale, in which 1 (one) corresponds to “Completely incorrect” and 5 (five) to “Completely correct”. In this study, the version translated and adapted for the Portuguese population was used [41]. Internal consistency, assessed using McDonald’s omega, was 0.92.

Mental Help Seeking Intention Scale—MHSIS [42]

The scale comprises three items, with the following wording: 1. If I were to experience a mental health problem, it would be my intention to seek help from a mental health professional (a more general and hypothetical intention, in the event of potentially developing a mental health problem in the future); 2. If I became concerned about a mental health problem, I would try to seek help from a mental health professional (subjectively perceiving concern or the initial recognition of symptoms, approaching a state of greater emotional awareness); 3. If a mental health concern or problem arose, I would plan to seek help from a mental health professional (the more deliberative and planned component of help-seeking behaviour, reflecting more structured preparation and intention to act).

Response options differ across items. Item 1 ranges from 1 (extremely unlikely) to 7 (extremely likely). For item 2, responses range from 1 (Definitely false) to 7 (Definitely true), while item 3 ranges from 1 (Strongly disagree) to 7 (Strongly agree). These different verbal anchors were retained from the original version [42]. All three items are rated on 7-point scales in the same direction, with higher values indicating a stronger intention to seek help from a mental health professional; no item requires reverse scoring. The total score was computed as the sum of the three items (range 3–21), which is a linear transformation of the mean item score used in the original version [42] and therefore yields equivalent results.

The instrument was translated and culturally adapted for use in Portugal as part of this study, following internationally recommended guidelines for the cross-cultural adaptation of self-report measures. The process involved six steps: (i) two independent forward translations into European Portuguese, carried out by native Portuguese speakers fluent in English, one with and one without health-related training; (ii) synthesis of the two translations into a single version, with discrepancies resolved by consensus between the translators and the researchers; (iii) two independent back-translations into English, carried out by native English speakers familiar with the Portuguese language and culture; (iv) synthesis of the back-translations and comparison with the original version, to ensure conceptual, semantic and linguistic equivalence; (v) review of the resulting version by a panel of experts in mental health, to assess content validity; and (vi) a pre-test with nursing students, to assess the comprehensibility and acceptability of the items. Construct validity was assessed through principal component analysis (PCA) conducted on the study sample (N = 388), using the Kaiser criterion (eigenvalues greater than 1) for component retention; as only one component was extracted, no rotation was required. The Kaiser–Meyer–Olkin measure of sampling adequacy was 0.75, indicating good adequacy of the data for this analysis, and Bartlett’s test of sphericity was statistically significant (χ^2^(3) = 1087.28, *p* < 0.001). The PCA supported a unidimensional structure, identifying a single component with an eigenvalue greater than 1 (eigenvalue = 2.68) that explained 89.33% of the total variance, with component loadings ranging from 0.92 to 0.96 and communalities from 0.85 to 0.92. Regarding reliability, the omega coefficient was 0.94. No items were modified or excluded during the adaptation process. Given the conceptual and linguistic equivalence achieved, the clear unidimensional structure, the high component loadings, and the high internal consistency, which replicate the properties reported for the original scale [42], the Portuguese version was considered adequate for use in the present analyses. Importantly, PCA is a data-reduction technique that summarises the total variance of the items and, unlike exploratory factor analysis, it does not model the shared variance attributable to latent factors. These results therefore provide only preliminary evidence of the dimensionality of the Portuguese version of the MHSIS, and its factorial structure requires confirmation through confirmatory factor analysis in an independent sample.

Positive Mental Health Questionnaire—+MHQ [17,18]

The +MHQ was developed by Lluch [18] and has already undergone a translation and adaptation process for the Portuguese population by Sequeira and colleagues [43]. The items form a multidimensional structure comprising the following factors:

Factor 1: Personal Satisfaction comprises items 4, 6, 7, 12, 14, 31, 38, and 39, with a maximum score of 32 points. This dimension assesses aspects related to self-concept and self-esteem, satisfaction with personal life, and the presence of an optimistic outlook regarding the future. It reflects the degree of personal self-worth, subjective well-being (SWB), and positive perceptions of one’s own life (internal consistency measured by the omega coefficient = 0.88);

Factor 2: Prosocial Attitude comprises items 1, 3, 23, 25, and 37, with a maximum score of 20 points. This factor assesses an active predisposition towards social and community involvement, including attitudes of helping, supporting, and accepting others. It also encompasses altruistic behaviours and the ability to accept different social and interpersonal realities (internal consistency measured by the omega coefficient = 0.79);

Factor 3: Self-Control comprises items 2, 5, 21, 22, and 26, totalling 20 points. This dimension assesses the ability to cope with stressful and conflict situations, as well as emotional balance and control. It also includes tolerance of frustration, anxiety, and stress, reflecting emotional self-regulation and psychological adaptation skills (internal consistency measured by the omega coefficient = 0.90);

Factor 4: Autonomy includes items 10, 13, 19, 33, and 34, with a maximum score of 20 points. It assesses the ability to develop one’s own criteria, act independently, and self-regulate behaviour. It also covers personal security, self-confidence, and the ability to make decisions autonomously (internal consistency measured by the omega coefficient = 0.81);

Factor 5: Problem-Solving and Personal Achievement comprises items 15, 16, 17, 27, 28, 29, 32, 35, and 36, with a maximum score of 36 points. This dimension assesses analytical and problem-solving skills, decision-making, flexibility, and adaptation to change. It also includes attitudes oriented towards personal growth, continuous development, and the achievement of personal goals (internal consistency measured by the omega coefficient = 0.77);

Finally, Factor 6: Interpersonal Relationship Skills comprises items 8, 9, 11, 18, 20, 24, and 30, totalling 28 points. This factor assesses relational skills, namely the ability to establish and maintain satisfactory interpersonal relationships, empathy, emotional understanding of others, and the ability to provide emotional support. It also reflects the ability to develop intimate and meaningful interpersonal relationships (internal consistency measured by the omega coefficient = 0.86).

### 2.5. Statistical Analysis

Data were analysed using IBM SPSS Statistics (version 31). First, the sample was described using descriptive statistics for the study variables (e.g., means, standard deviations, and coefficients of variation), complemented by an assessment of the shape of the distributions based on critical skewness and kurtosis ratios. Because critical ratios become inflated in large samples, raw skewness and kurtosis values were also examined; absolute values below 2 and 7, respectively, being considered indicative of no severe departure from normality [44,45]. Ceiling effects were quantified as the percentage of participants obtaining the maximum possible score on each total score, with values above 15% being considered indicative of a ceiling effect [46].

The association between close contact with mental illness (family members or close friends) and personal experience of a mental health problem was examined using the chi-squared test, complemented by the calculation of Cramér’s V as a measure of association.

To examine the effects of year of study and familiarity with mental illness on the overall scores of the instruments, a 2 × 2 factorial MANOVA was conducted. For this analysis, familiarity was operationalised as a dichotomous variable: students who answered affirmatively to both familiarity items (close contact and personal experience) were classified as having high familiarity (n = 117) and were compared with all remaining students (n = 271). Partial eta squared was used as a measure of effect size, both for the multivariate tests and for the subsequent univariate tests. Univariate follow-up tests, which were not planned a priori, were conducted for the significant multivariate effects and interpreted using a Bonferroni-adjusted significance level (α = 0.05/5 = 0.01) to control for type I error inflation.

The assumptions underlying the analyses were assessed beforehand. Specifically, multivariate normality was indirectly assessed using the distribution of each dependent variable; the homogeneity of the variance-covariance matrices was assessed using Box’s M test; and the homogeneity of variances was assessed using Levene’s test. Pillai’s Trace was selected as the primary multivariate statistic because it is more robust to potential violations of these assumptions in designs with unequal group sizes. Given the sample size and cell sizes (n = 57 to 138) and the fact that skewness was in the same (negative) direction across groups, the parametric procedures were considered sufficiently robust to the departures from normality observed [47]. Nevertheless, as a sensitivity analysis, the univariate group comparisons were replicated using Mann–Whitney U tests. These were interpreted using the same Bonferroni-adjusted threshold (α = 0.01), and effects whose significance differed between the parametric and nonparametric analyses were considered less robust and interpreted with caution.

The relationship between the set of MHL dimensions (MHLS, MHKS, and MHPK) and the set of PMH dimensions (+MHQ) was explored using canonical correlation analysis. Wilks’ test was used to assess the significance of the successive extracted functions, and redundancy indices were used to complement the interpretation of the practical magnitude of the association found, in addition to the canonical correlation coefficient.

Finally, to identify factors associated with HSI, hierarchical multiple regression was performed using the Enter method, in three successive blocks. Prior to the analysis, the assumptions of linearity and homoscedasticity of the relationship between the predictors and the dependent variable, normality of the residual distribution, independence of errors using the Durbin–Watson statistic, and absence of multicollinearity among the predictors based on tolerance values and the variance inflation factor (VIF) were assessed.

### 2.6. Ethical Considerations

This study was conducted in accordance with the principles outlined in the Declaration of Helsinki for research involving human subjects. The study was approved by the Ethics Committee of the Health Sciences Research Unit: Nursing (UICISA: E) of the Nursing School of Coimbra University [ESEUC]. All participants remained anonymous, and data were stored in a secure, encrypted online archive accessible only to the study’s authors. The survey included an electronic informed consent form. The consent form also included permission to publish the research data.

## 3. Results

### 3.1. Sample Characteristics

The final sample comprised 388 students enrolled in the second (n = 195; 50.26%) and fourth (n = 193; 49.74%) years of the Bachelor’s Degree in Nursing, of whom 84.0% (n = 326) are female and 16.0% (n = 62) are male.

Participants’ ages ranged from 19 to 53 years, with a mean age of 21.76 years (SD = 5.00 years; median = 21.0; n = 387, one missing value), indicating that the sample was predominantly young, consistent with a university population at the beginning of their academic training (1st cycle).

For mothers’ educational attainment, the most frequent level was upper secondary education (39.18%), followed by a bachelor’s degree (27.84%) and lower secondary education (12.89%). Higher education qualifications were reported for 35.31% of mothers, indicating a relatively differentiated socio-educational profile. Only one participant selected the “not applicable” option (0.26%).

For fathers’ educational attainment, the distribution was slightly different. Upper secondary education remained the most frequent level (31.70%), but lower secondary education accounted for a higher proportion than among mothers (22.16%). The proportion with higher education qualifications was lower among fathers: 19.07% held a bachelor’s degree and 4.90% held a master’s or doctoral degree, totalling 23.97%, compared with 35.31% among mothers. The “not applicable” category had a slightly higher frequency for fathers (2.06%).

### 3.2. Personal and Interpersonal Familiarity with Mental Illness

Familiarity with mental health problems was assessed through close contact (family members or close friends) and personal experience. More than half of the students (52.6%; n = 204) reported having family members or close friends who had experienced mental health problems, whereas 47.4% (n = 184) reported no such contact, indicating considerable interpersonal contact with mental health problems in this sample.

Among students with such contacts, 37.6% reported that the person concerned had received care from healthcare professionals, 25.8% reported that they had not sought professional help, and 36.6% did not know whether specialised care had been received. This high proportion of uncertainty may reflect limited awareness of the care pathways followed by close contacts or, alternatively, the social invisibility of mental health treatment.

Among the professionals consulted, psychologists were the most frequently reported (38.9%), followed by a combination of a physician/psychiatrist and a psychologist (21.9%), psychiatrists (20.4%), and family physicians (19.8%). Across these categories, a high proportion of students rated the care provided as “neither helpful nor harmful” (41.0% to 58.5%), which may reflect limited information about the outcomes of the care received by their close contacts.

With regard to treatment, 31.2% of students reported that their close contacts had received non-pharmacological interventions, such as psychotherapy or regular follow-up, 29.1% reported medication, and 24.2% reported a combination of both. Again, a high proportion of students did not know which treatments had been received (40.5% to 43.8%).

With regard to personal experience, 37.6% of students (n = 146) reported personal experience of a mental health problem. This indicates that personal experiences of mental health problems are common in this sample, although these data do not constitute an estimate of clinical prevalence. Among these students, the psychologist was the most frequently consulted professional (27.1%), followed by the psychiatrist (10.1%), the physician (9.8%), and the nurse (6.2%), suggesting a greater use of psychological support than of other types of specialised care.

The association between close contact and personal experience was statistically significant (χ^2^_(1)_ = 71.31, *p* < 0.001, Cramér’s V = 0.43). Overall, 30.2% of students (n = 117) answered affirmatively to both items (high familiarity), 22.4% (n = 87) reported only close contact, and 7.5% (n = 29) reported only personal experience, whereas 39.9% (n = 155) reported neither. For the MANOVA, familiarity was operationalised as an affirmative answer to both items (n = 117, 30.2%), compared with all remaining students (n = 271, 69.8%).

### 3.3. Descriptive Statistics of the Study Variables

Regarding the summary statistics for instrument scores (Table 1), the coefficients of variation were generally moderate (9.27% to 23.16%), indicating low to moderate relative dispersion around the means, with the exception of the MHSIS (CV = 23.16%) and the self-control dimension of the +MHQ (CV = 20.21%), which showed greater relative variability. The total scores of the three instruments (MHLS, +MHQ) presented lower coefficients of variation than those of their respective dimensions.

An examination of the distribution shapes, based on the critical skewness and kurtosis ratios, revealed a differentiated pattern between the dimensional level and the total scores.

With the exception of available professional help, interpersonal relationship skills, and self-control, most of the dimensions of the MHLS and +MHQ showed deviations from symmetry (|z| > 1.96), with a predominance of negative skewness, suggesting a tendency for responses to cluster at the higher values of the scale (ceiling effect). This pattern was substantially attenuated at the level of the total MHLS scores.

The total MHKS was the most notable exception, with markedly high critical skewness and kurtosis ratios (As = −11.40; Ks = 23.43), indicating a pronounced deviation from normality. However, the raw skewness and kurtosis values of all total scores were within acceptable limits (skewness from −1.82 to −0.32; kurtosis from −0.15 to 5.79), with the MHKS showing the highest kurtosis (Sk = −1.41; Ku = 5.79). No participant obtained the maximum possible score on the MHKS (observed maximum = 58 of 60), suggesting that its deviation from normality reflects a long left tail of low scores rather than an accumulation of responses at the maximum. With regard to ceiling effects in the total scores, no participant obtained the maximum possible score on the MHKS, +MHQ or MHLS total score (and only 0.8% on the corrected attitudes dimension), whereas 39.7% of participants obtained the maximum score on the MHPK-10 and 20.4% on the MHSIS, indicating a ceiling effect on these two measures.

### 3.4. Familiarity × Year of Study in MHL and PMH: Factorial MANOVA 2 × 2

To test the effect of year of study (second vs. fourth year) and familiarity with mental illness (high familiarity, n = 117, vs. remaining students, n = 271) on the overall scores of the instruments (MHLS, MHPK-10, MHKS, +MHQ and MHSIS), a 2 × 2 factorial MANOVA was conducted (cell sizes from 57 to 138). Box’s test indicated heterogeneity of the covariance matrices (Box’s M = 87.92, F(45, 144,068.50) = 1.90, *p* < 0.001); therefore, Pillai’s trace, which is the most robust multivariate statistic for this violation, was used. Levene’s tests indicated homogeneity of error variances for all dependent variables except the MHPK-10 (based on the mean, *p* < 0.001; based on the median, *p* = 0.030), whose univariate results should therefore be interpreted with caution.

The multivariate tests revealed statistically significant but small main effects of familiarity with mental illness (Pillai’s Trace = 0.048, F(5, 380) = 3.833, *p* = 0.002, η^2^p = 0.048) and of year of study (Pillai’s Trace = 0.043, F(5, 380) = 3.419, *p* = 0.005, η^2^p = 0.043) on the set of overall scale scores, whereas the year of study × familiarity interaction was not significant (Pillai’s Trace = 0.018, F(5, 380) = 1.408, *p* = 0.220, η^2^p = 0.018).

The significant multivariate main effects were followed up with univariate ANOVAs, which were interpreted using a Bonferroni-adjusted significance level to control for multiplicity (α = 0.05/5 = 0.01). A univariate interaction effect was observed only for the MHSIS (F(1, 384) = 4.342, *p* = 0.038, η^2^p = 0.011), which did not survive Bonferroni correction (all other *p* ≥ 0.306).

The multivariate effect of familiarity was mainly attributable to the +MHQ (F(1, 384) = 15.930, *p* < 0.001, η^2^p = 0.040), an effect that remained significant after Bonferroni correction, with students with high familiarity (n = 117) presenting lower mean scores than the remaining students (n = 271) (118.4 vs. 125.3), both in the second year (118.4 vs. 125.2) and in the fourth year (118.4 vs. 125.5). Familiarity was not associated with differences in the remaining measures (all *p* ≥ 0.099).

The multivariate effect of year of study was mainly attributable to the MHSIS, with fourth-year students presenting higher help-seeking intention than second-year students (16.6 vs. 15.6; F(1, 384) = 10.585, *p* = 0.001, η^2^p = 0.027), an effect that remained significant after Bonferroni correction. Fourth-year students also presented higher mean scores on the MHLS total score (124.1 vs. 121.0; F(1, 384) = 5.648, *p* = 0.018, η^2^p = 0.014) and the MHPK-10 (46.3 vs. 44.8; F(1, 384) = 6.480, *p* = 0.011, η^2^p = 0.017), but these effects did not remain significant after Bonferroni correction. No differences by year of study were found for the MHKS or the +MHQ (all *p* ≥ 0.414). The univariate interaction for the MHSIS indicated that, among second-year students, those with high familiarity reported lower help-seeking intention than the remaining students (14.5 vs. 16.1), whereas no such difference was observed among fourth-year students (16.7 vs. 16.5); as neither the multivariate interaction nor the corrected univariate effect was significant, this pattern should be regarded as exploratory. All effect sizes were small (Table 2).

Sensitivity analyses using Mann–Whitney U tests confirmed the higher scores of fourth-year students on the MHLS total score (Z = −2.94, *p* = 0.003) and the MHSIS (Z = −2.69, *p* = 0.007), both below the Bonferroni-adjusted threshold. The two approaches therefore differed regarding the MHLS: this difference did not survive correction in the parametric analysis but reached the adjusted threshold in the nonparametric analysis, and it was therefore considered less robust. In contrast, the difference in the MHPK-10 was not replicated (Z = −1.49, *p* = 0.135), possibly reflecting the ceiling effect and the heterogeneity of variances observed in this measure, and a nominal difference emerged for the MHKS (Z = −2.09, *p* = 0.036) that did not survive correction. The effect of familiarity on the +MHQ was confirmed (Z = −4.50, *p* < 0.001). No other differences were found (all *p* ≥ 0.219).

### 3.5. MHL and PMH: Canonical Correlation

The CCA extracted six canonical functions (Table 3), of which only the first revealed a statistically significant association between the MHL set (dimensions of the MHLS, MHKS, and MHPK) and the PMH set (+MHQ dimensions), with Rc = 0.423, Wilks’ λ = 0.730, F(48, 1844.30) = 2.537, *p* < 0.001, explaining approximately 17.9% of the variance shared between the two canonical variates (r^2^c = 0.179).

Analysis of the canonical loadings for Function 1 (Table 4) shows that, on the mental health literacy side, the most substantial contributions (considering a relevance criterion of |0.30| or higher) come from attitudes that promote recognition and help-seeking (0.903), knowledge of factors that promote good mental health (MHPK; 0.656), and knowledge of how to seek information (0.399). The remaining indicators, including the ability to recognise mental health problems (0.292), general mental health knowledge (MHKS; 0.203), knowledge of available professional help (0.129), knowledge of causes and risk factors (0.035), and knowledge of self-help strategies (0.011), showed low loadings. This suggests that the attitudinal component of literacy, together with knowledge of the determinants of good mental health, is primarily responsible for structuring this association.

On the positive mental health side, the most substantial contributions came from prosocial attitude (0.882), problem-solving and personal achievement (0.772), and interpersonal relationship skills (0.666), whereas self-control (0.266), personal satisfaction (0.260), and autonomy (0.019) showed low loadings. This pattern indicates that Function 1 mainly captures the prosocial and relational facets of positive psychological functioning, together with problem-solving and personal achievement.

Overall, the results suggest that students with more favourable attitudes towards recognition and help-seeking and greater knowledge of the determinants of good mental health tend to report higher levels of positive mental health, particularly with regard to prosocial orientation, personal achievement, and interpersonal skills.

However, it is important to consider the practical magnitude of the reported association. The redundancy indices show that Function 1 explains only 3.5% of the variance in the MHL set from PMH and 5.8% of the variance in PMH from MHL, values considerably lower than the variance explained by each function within its own set (19.4% and 32.6%, respectively).

Therefore, the statistical significance of the function should not be interpreted as evidence of substantial overlap between the two constructs, but rather, in our view, as a modest association pattern restricted to specific components of both constructs.

### 3.6. Hierarchical Multiple Regression Analysis Predicting Help-Seeking Intention

To test the relative contribution of MHL, including the MHKS and MHPK, and PMH in predicting mental health help-seeking intention, while controlling for the effects of year of study and familiarity with mental illness, a hierarchical multiple regression analysis (Enter method) was conducted in three successive blocks.

In the first block (Table 5), year of study and familiarity with mental illness were entered; in the second block, the six MHLS dimensions, MHKS scores, and MHPK scores were entered; and in the third block, the six dimensions of positive mental health were entered. This sequence made it possible to assess the increase in explained variance (ΔR^2^) associated with each block, isolating the specific contribution of MHL and PMH relative to year of study and familiarity with mental illness.

In the first model, year of study and familiarity with mental illness explained a small but statistically significant proportion of the variance in HSI (R = 0.154; R^2^ = 0.024; adjusted R^2^ = 0.019; F(2, 385) = 4.69, *p* = 0.010), driven by year of study (b = 0.99, β = 0.13, *p* = 0.009), whereas familiarity with mental illness was not a significant predictor (*p* = 0.106).

The introduction of the MHL variables in the second model significantly increased the explained variance (ΔR^2^ = 0.096; ΔF(8, 377) = 5.14, *p* < 0.001; R^2^ = 0.120; adjusted R^2^ = 0.096). Attitudes that promote recognition (β = 0.21, *p* < 0.001) and knowledge of ways to seek information (β = 0.13, *p* = 0.011) emerged as significant predictors, whereas knowledge of causes and risk factors was marginal (β = 0.10, *p* = 0.061) and year of study remained significant (β = 0.10, *p* = 0.046). The introduction of the six PMH dimensions in the third model further increased the explained variance (ΔR^2^ = 0.043; ΔF(6, 371) = 3.15, *p* = 0.005), resulting in a final model with R = 0.403, R^2^ = 0.162 and adjusted R^2^ = 0.126 (F(16, 371) = 4.50, *p* < 0.001). The final model therefore explained 16.2% of the variance in help-seeking intention in the sample (R^2^ = 0.162) and, after adjustment for the number of predictors, approximately 12.6% (adjusted R^2^ = 0.126), which represents a modest explanatory contribution, with most of the variance remaining unexplained by the variables considered.

In the final model, attitudes that promote recognition (β = 0.17, *p* = 0.004), problem-solving and personal achievement (β = 0.18, *p* = 0.015), knowledge of causes and risk factors (β = 0.11, *p* = 0.036), year of study (β = 0.11, *p* = 0.033), and interpersonal relationship skills (β = 0.12, *p* = 0.046) remained significant and independent positive predictors, all with small effect sizes. Problem-solving and personal achievement and attitudes that promote recognition showed the largest standardised coefficients in the model.

It should be noted that knowledge of ways to seek information, which was significant at the second step, was reduced to marginal significance after the introduction of the positive mental health dimensions (β = 0.09, *p* = 0.082), suggesting shared variance between informational literacy and psychological resources.

With regard to model adequacy, there was no evidence of multicollinearity (tolerance values between 0.40 and 0.94; VIF values between 1.07 and 2.49). The Durbin–Watson statistic (1.94) indicated independence of errors. Standardised residuals ranged from −3.64 to 2.13, with only three cases (0.8%) exceeding |3|, which is within the expected range for a sample of this size; visual inspection of the scatterplot of standardised residuals against standardised predicted values and of the normal P–P plot showed no relevant departures from linearity, homoscedasticity, or normality of residuals.

## 4. Discussion

The findings allow an integrated interpretation in response to the four objectives presented at the end of the Introduction, revealing a pattern consistent with the recent literature on this population, namely nursing students, while highlighting nuances that challenge some common interpretations regarding the relationship between these constructs.

### 4.1. Familiarity with Mental Illness: Exposure Without Instrumental Literacy

In this sample, substantial personal and interpersonal contact with mental illness was observed. More than half of the students reported that their family members or close friends had mental health problems. More than one third reported having personally experienced a mental health problem. The two forms of familiarity were moderately associated, and approximately three in ten students reported both, which defined the “high-familiarity” group used in the group comparisons.

Before interpreting these findings further, it is important to clarify how the variables in question were operationalised. These are dichotomous variables (yes/no) that do not capture the quality of the exposure, as the “yes” response may encompass very different experiences in terms of duration, type of involvement, intensity of contact, emotional burden, etc. Thus, although students reported having contact with mental illness, a high proportion did not know whether the person close to them had sought professional help (36.6%) and were unaware of the type of treatment received (40.5% to 43.8%), suggesting that a substantial proportion of this contact may be limited to awareness and information rather than to an emotional closeness that could influence students’ instrumental literacy, as reported in the literature [9,10].

The finding that more than one third of students reported having experienced mental health problems is consistent with the literature on the mental health of higher education students, which documents a high burden of psychological distress in this population and, in many cases, help-seeking that falls short of identified needs [1].

### 4.2. Mental Health Literacy, Positive Mental Health, and Help-Seeking Intention Profile

The response pattern found across most dimensions of MHL, PMH, and mental health help-seeking intention is generally characterised by negative skewness and a concentration of responses at the higher values of the scales and subscales, which is consistent with a population particularly sensitised to these topics through the curriculum. However, second-year students are at an earlier stage of the programme, without formal training in psychiatry and mental health, and the comparison between years of study (Section 3.4) did not reveal significant differences across most of these dimensions, with the exception of a small difference in help-seeking intention, which remained significant after correction for multiple comparisons, and small differences in the MHLS total score and MHPK-10, which did not.

Together, these observations suggest that curricular training alone may not account for the overall ceiling pattern observed here. One possible, albeit speculative, explanation is a self-selection effect into nursing, as documented in the systematic review by Wu and colleagues [48] on the factors influencing programme choice among health sciences students, in which prosocial values, a care orientation, and previous personal or family experiences with illness emerge as strong motivations for choosing the programme. This possibility could not be tested in the present study and requires longitudinal confirmation.

This possibility is consistent with longitudinal studies that question whether programme choice reflects early socialisation or a pre-existing predisposition [49].

This interpretation does not, however, rule out a possible contribution from the curriculum. A study with a design comparable to ours found higher general health literacy among 4th-year than among 1st-year students of a nursing programme [50], which is consistent with, although not evidence of, a role of the curriculum in some dimensions of health literacy.

The MHKS results, whose deviation is more pronounced than that of any other measure, warrant further consideration. Since no participant obtained the maximum possible score, this pattern does not reflect a ceiling effect in the strict sense, but rather a strong concentration of responses in the upper range of the scale together with a small number of very low scores; it may also result from social desirability. Furthermore, the Likert and Likert-type response items used in these instruments, including the MHKS, do not assess declarative knowledge but rather a form of self-assessment of what respondents believe they know, which is closer to the concept of metacognition than to objective knowledge [51].

Within the field of MHL, the scoping review by Wei and colleagues specifically distinguishes between instruments that assess factual declarative knowledge using true/false/don’t know formats or recognition of disorders based on vignettes, and instruments that constitute self-assessment measures of the extent of one’s knowledge, a category that includes scales such as the MHLS and, to some extent, the MHKS [52].

### 4.3. Differences by Year of Study and Familiarity in MHL, PMH, and Mental Health Help-Seeking Intention

The small multivariate main effects of year of study and familiarity on the set of overall measures, together with the absence of interaction effects and the fact that only the univariate effects of familiarity on PMH and of year of study on help-seeking intention remained significant after Bonferroni correction, indicate that year of study and familiarity with mental illness are associated with only small and circumscribed differences in well-being and help-seeking intention in these students. Given the cross-sectional design, these between-group differences cannot be attributed to progression through the programme, as cohort effects, selection effects, and pre-existing differences between students cannot be ruled out.

The small between-year differences in the MHLS total score, MHPK-10 and MHSIS, with fourth-year students reporting higher scores than second-year students, are consistent with, although not evidence of, an influence of curricular exposure to mental health and psychiatry content during the 3rd year.

Of these differences, only the higher help-seeking intention of fourth-year students remained significant after Bonferroni correction, and it was also supported by the nonparametric sensitivity analysis; the differences in the MHLS total score and MHPK-10 should therefore be regarded as exploratory. The MHLS difference, although not significant after correction in the parametric analysis, reached the adjusted threshold in the nonparametric analysis (*p* = 0.003), whereas the difference in the MHPK-10 was not replicated, which may be related to the ceiling effect and the heterogeneity of variances observed in the latter. If confirmed in future longitudinal studies, this pattern would be consistent with the notion that formal instruction and direct clinical contact with people experiencing mental illness, as opposed to informal and unmediated contact (personal and interpersonal contact), are associated with modestly higher knowledge about mental health promotion and willingness to seek professional help.

Regarding familiarity, students who reported both close contact with and personal experience of mental health problems reported lower positive mental health than the remaining students, a difference that remained significant after Bonferroni correction and was consistent across years of study, although its magnitude was small (η^2^p = 0.040). Because the high-familiarity group, by definition, includes personal experience of a mental health problem, this difference may reflect the students’ own current or past mental health difficulties rather than an effect of contact with mental illness itself. This interpretation is consistent with the dual-continuum model [15], according to which mental illness and positive mental health are related but distinct dimensions. The cross-sectional design and the dichotomous operationalisation of familiarity do not allow these explanations to be disentangled.

The univariate interaction observed for help-seeking intention, whereby second-year students with high familiarity reported lower intention than their peers, a difference not observed among fourth-year students, did not survive correction for multiple comparisons and was not supported at the multivariate level. It is nevertheless noteworthy that this pattern emerged among students who had not yet received formal training in mental health, and it deserves examination in future studies, namely regarding the role of self-stigma and the anticipated consequences of disclosure [5,25].

Familiarity was not associated with differences in MHL, mental health knowledge or help-seeking intention, and the effects detected were small; their practical relevance to educational practice should be interpreted with caution. These findings should not be regarded as evidence that year of study or familiarity constitute determinants of these constructs.

### 4.4. The Relationship Between Mental Health Literacy and Positive Mental Health

Canonical correlation analysis isolated only one significant canonical function, indicating a modest association (Rc = 0.423; r^2^c = 0.179) between the MHL set and the PMH set, with the redundancy indices showing that the overlap in variance between the two constructs is considerably smaller than the canonical correlation coefficient alone would suggest (3.5% and 5.8%, depending on the direction considered).

This finding converges with the available evidence on the relationship between MHL and well-being, which has identified associations of negligible magnitude [26], and is also consistent with the study by Gorczynski and colleagues among British university students, which found no relationship between MHL and well-being [34]. However, it diverges from interpretations that attribute a more consistent relationship to MHL and PMH [35].

The pattern of canonical loadings for the function allows us to establish that it is not MHL as a whole that is associated with PMH, but specifically its attitudinal component (attitudes that promote recognition and help-seeking) and knowledge about the factors that promote good mental health (MHPK) and, to a lesser extent, knowledge of how to seek information. In contrast, the components related to knowledge about mental disorders (recognition of disorders, causes and risk factors, self-help strategies, available professional help, and general mental health knowledge) contributed little to this function.

This dissociation between the different components of MHL in their relationship with PMH is consistent with our earlier observation that MHL should not be understood as a unitary and homogeneous construct, but rather as a configuration of partially overlapping and functionally distinct components [13,21], reinforcing the notion that MHL has undergone a conceptual expansion that does not always find solid empirical correspondence in the way its different components relate to one another and to neighbouring constructs, such as PMH [20].

These findings suggest that favourable attitudes towards recognition and help-seeking, together with knowledge of what promotes mental health, share a proportion of their variance with positive psychological functioning, particularly its prosocial and relational facets, whereas knowledge about mental disorders appears to operate relatively independently of well-being.

### 4.5. Predictors of Help-Seeking Intention

The final hierarchical model explained 16.2% of the variance in mental health help-seeking intention in the sample (R^2^ = 0.162) and approximately 12.6% after adjustment for the number of predictors (adjusted R^2^ = 0.126), the latter providing a more conservative estimate of the variance expected to be explained in the population. This is a modest explanatory contribution, indicating that most of the variance in help-seeking intention depends on factors not included in the model; the predictors identified should therefore not be regarded as a comprehensive explanation of this outcome. This modest proportion is consistent with the weak association between MHL and HSI previously described in the meta-analysis by Özparlak and colleagues [26], as well as with the theoretical framework of the Theory of Planned Behaviour, according to which help-seeking intention depends on a broader set of determinants, including subjective norms and perceived behavioural control, variables that were not included in the present model [22,23].

Among the MHL predictors, attitudes that promote recognition and help-seeking and knowledge of causes and risk factors stood out, both showing positive associations with HSI.

Theoretically, the positive association between attitudes and HSI is consistent with the Theory of Planned Behaviour, according to which more favourable attitudes towards a behaviour translate into a greater intention to perform it [22,23].

Among these students, more favourable attitudes towards recognising mental health problems and seeking help were associated with a greater intention to seek professional help, even after controlling for knowledge and PMH. Nevertheless, the literature on stigma among nursing students has documented that favourable general attitudes do not always translate into personal help-seeking, a phenomenon described as an attitude–behaviour gap [6,7].

Previous research suggests that, in this professional group, recognising one’s own psychological vulnerability may be perceived as incompatible with the developing professional identity, potentially fostering self-stigma and a withdrawal from formal help-seeking [5,25]. However, as professional identity and self-stigma were not measured in the present study, these remain hypotheses to be tested in future research rather than mechanisms supported by our data.

This relationship between MHL and HSI has also been described in qualitative studies showing concerns about exposure and a preference for resolving problems independently [5].

Problem-solving and personal achievement and interpersonal relationship skills, both dimensions of PMH, emerged as significant predictors and showed larger effect magnitudes than most MHL predictors, with the former showing the largest standardised coefficient in the model, although all effects were small.

This finding suggests that positive psychological functioning resources, particularly the ability to identify and mobilise solutions in the face of adversity and the competence to establish supportive interpersonal relationships, may play a role in help-seeking intention comparable to that of knowledge about mental health, although the small effect sizes and the modest proportion of variance explained preclude stronger conclusions.

### 4.6. Limitations

This study has several limitations. First, the cross-sectional design precludes causal inference regarding the relationships identified. This applies both to the interpretation of the differences by year of study and familiarity, which may reflect cohort effects, selection effects, or pre-existing differences rather than the influence of the programme, and to the relationships between MHL, PMH, and HSI. Longitudinal designs following the same students throughout the programme are needed to examine change over time.

Second, the sample was recruited from a single higher education institution, which limits the generalisability of the findings to other educational and institutional contexts. In addition, 84.0% of the students were female. Although this gender disparity is common among nursing students, it precludes an analysis of potential gender differences, which are widely documented in the literature on mental health help-seeking. With only 62 male students, distributed across the four year of study × familiarity groups, subgroup or sensitivity analyses by gender would have very low statistical power and unstable estimates and were therefore not conducted. Given that women tend to report higher mental health literacy and more favourable help-seeking attitudes and intentions, the predominance of female participants may have contributed to the high scores observed, and the findings may not be generalisable to male nursing students. Future studies should use sampling strategies that ensure adequate representation of men.

Third, approximately 38% of eligible students did not participate in the study, and no information was available on the characteristics of non-participants, which precluded a comparison with participants. Because participation was voluntary, non-response and volunteer (self-selection) bias cannot be ruled out: students more interested in or sensitised to mental health issues, or with more favourable attitudes towards help-seeking, may have been more likely to participate. This may have contributed to the high scores and negatively skewed distributions observed and may have attenuated differences between years of study. Nevertheless, participation rates were very similar in the second and fourth years (62.17% and 61.21%, respectively), which suggests that differential non-response between years is unlikely to account for the comparisons performed.

Furthermore, the MHSIS, the measure of HSI used in this study, was translated and validated as part of the study itself. Although its psychometric properties in the present sample were favourable (a single component in the PCA and high internal consistency), PCA is a data-reduction technique rather than a latent variable model and provides only preliminary evidence of dimensionality; confirmatory factor analysis in an independent sample is therefore required to confirm its factorial structure and reliability. In addition, the same sample was used both for the psychometric analysis of the MHSIS and for hypothesis testing, which may have led to an overestimation of its psychometric adequacy.

Finally, reliance on self-report measures makes the results susceptible to potential social desirability bias, particularly in the items assessing attitudes, as well as to the limitations of the operationalisation of familiarity with mental illness, which does not capture the type, intensity, or nature of the contact. Moreover, for the group comparisons, familiarity was dichotomised as high familiarity (both close contact and personal experience) versus all remaining students, which resulted in unequal group sizes and grouped students with only one type of familiarity together with those with none. In addition, a ceiling effect was observed for the MHPK-10 (39.7% at the maximum score) and the MHSIS (20.4%), which may have limited the ability of these measures to discriminate between students with higher levels of knowledge or help-seeking intention and may have attenuated the associations and differences observed. Although the sample size supports the robustness of the parametric procedures used, and the main findings were replicated in nonparametric sensitivity analyses, the non-normal distribution of some variables should be considered when interpreting the results. Finally, relevant determinants of help-seeking intention, such as professional identity, self-stigma, subjective norms, and perceived behavioural control, were not measured, and their potential role should be examined in future studies.

### 4.7. Implications for Nursing Education

These findings suggest that educational initiatives targeting nursing students in the field of mental health could benefit from going beyond the transmission of knowledge about mental disorders and factors that promote mental health, and from also fostering favourable attitudes towards help-seeking and positive psychological functioning resources, particularly problem-solving and interpersonal relationship skills. However, as these implications derive from cross-sectional associations, the effectiveness of such approaches has not been demonstrated by the present study. Future research should evaluate targeted interventions longitudinally, ideally including measures of actual help-seeking behaviour rather than intention alone.

## 5. Conclusions

This study indicates that mental health literacy, positive mental health, and help-seeking intention, although often treated as components of the same process, are only modestly related among nursing students, with associations that are component-specific, mainly involving attitudes that promote recognition and help-seeking and knowledge about mental health promotion.

Differences by year of study and familiarity were small: fourth-year students reported higher help-seeking intention than second-year students, and students with both close contact with and personal experience of mental health problems reported lower positive mental health. Given the cross-sectional design, these differences cannot be attributed to progression through the programme or to familiarity itself.

Favourable attitudes towards recognising mental health problems and seeking help were associated with a greater intention to seek help, supporting the inclusion of attitudinal components in educational interventions. The potential role of professional identity and self-stigma, which were not measured in this study, warrants future investigation.

Positive psychological functioning resources, particularly problem-solving and interpersonal skills, emerged as modest predictors of help-seeking intention, with effects comparable in magnitude to those of knowledge about mental health, although the model as a whole explained only a small proportion of the variance (R^2^ = 0.162; adjusted R^2^ = 0.126). These findings suggest that the education of these students might benefit from complementing the transmission of mental health knowledge with the development of internal resources that may support the decision to seek help, a possibility that should be tested in longitudinal and intervention studies.

## Figures and Tables

**Table 1:** Summary statistics of variables (N = 388).

Variables	Mean	SD	CV	As	Ks
Ability to recognise mental health problems	24.54	3.63	14.80	−4.09	6.23
Knowledge of how to seek information	15.40	2.48	16.07	−2.49	2.72
Knowledge of causes and risk factors	5.06	0.95	18.80	−2.31	6.97
Knowledge of self-help strategies	5.99	1.21	20.20	−3.28	2.05
Knowledge of available professional help	8.25	1.51	18.27	−1.56	2.57
Attitudes that promote recognition and self-help	63.29	8.09	12.78	−2.58	0.01
MHLS—total score	122.53	11.36	9.27	−2.73	1.71
MHKS—total score	44.55	5.85	13.14	−11.40	23.43
Personal Satisfaction	27.03	3.69	13.65	−7.52	3.85
Prosocial Attitude	17.93	1.88	10.47	−10.52	9.37
Self-Control	14.34	2.90	20.21	−0.60	−2.02
Autonomy	15.18	2.85	18.78	−4.01	0.36
Problem-Solving and Personal Achievement	28.14	4.67	16.59	−6.72	6.67
Interpersonal Relationship Skills	22.58	3.01	13.35	−1.85	−1.60
+MHQ—total score	123.24	16.00	12.98	−5.23	1.94
MHSIS—total score	16.11	3.73	23.16	−3.92	−0.60

**Table 2:** Univariate follow-up tests of the 2 × 2 factorial MANOVA: means (SD) by year of study and familiarity (N = 388).

Measure	Group 1, M (SD)	Group 2, M (SD)	F(1, 384)	*p*	η^2^p
Year of study: Group 1 = second year (n = 195); Group 2 = fourth year (n = 193)					
MHLS—total score	120.98 (11.47)	124.10 (11.06)	5.65	0.018	0.014
MHKS—total score	44.23 (4.99)	44.88 (6.61)	0.67	0.414	0.002
MHPK-10—total score	44.83 (6.98)	46.28 (5.19)	6.48	0.011	0.017
+MHQ—total score	123.20 (16.19)	123.28 (15.85)	0.01	0.935	0.000
MHSIS—total score	15.62 (3.73)	16.60 (3.68)	10.59	0.001 *	0.027
Familiarity: Group 1 = high familiarity (n = 117); Group 2 = remaining students (n = 271)					
MHLS—total score	122.65 (11.48)	122.48 (11.33)	0.01	0.933	0.000
MHKS—total score	44.14 (6.42)	44.73 (5.59)	0.85	0.357	0.002
MHPK-10—total score	45.28 (6.67)	45.67 (5.97)	0.39	0.532	0.001
+MHQ—total score	118.38 (14.70)	125.33 (16.11)	15.93	<0.001 *	0.040
MHSIS—total score	15.66 (3.99)	16.30 (3.60)	2.73	0.099	0.007

Note: Multivariate tests (Pillai’s trace): year of study, V = 0.043, F(5, 380) = 3.42, *p* = 0.005, η^2^p = 0.043; familiarity, V = 0.048, F(5, 380) = 3.83, *p* = 0.002, η^2^p = 0.048; year of study × familiarity, V = 0.018, F(5, 380) = 1.41, *p* = 0.220, η^2^p = 0.018. Univariate interaction effects: MHSIS, F(1, 384) = 4.34, *p* = 0.038, η^2^p = 0.011; all other *p* ≥ 0.306. * Significant at the Bonferroni-adjusted level (α = 0.01).

**Table 3:** Canonical Correlations and Significance Tests (N = 388).

Functions	Rc	Eigenvalue	λ de Wilks	F	Num D.F	Denom D.F.	*p*
1	0.423	0.218	0.730	2.537	48.00	1844.30	<0.001
2	0.235	0.059	0.889	1.279	35.00	1579.91	0.128
3	0.182	0.034	0.941	0.959	24.00	1312.92	0.520
4	0.131	0.018	0.973	0.680	15.00	1041.13	0.807
5	0.085	0.007	0.991	0.449	8.00	756.00	0.891
6	0.048	0.002	0.998	0.286	3.00	379.00	0.835

**Table 4:** Canonical solution of function 1 (Rc = 0.423; r^2^c = 0.179; *p* < 0.001).

Variables	Standardized Canonical Correlation Coefficients	Canonical Loading	Cross-Loading
Set 1—Mental Health Literacy			
Ability to recognise mental health problems	−0.001	0.292	0.124
Knowledge of how to seek information	0.199	0.399	0.169
Knowledge of causes and risk factors	−0.018	0.035	0.015
Knowledge of self-help strategies	−0.039	0.011	0.005
Knowledge of available professional help	0.065	0.129	0.055
Attitudes that promote recognition and self-help	0.773	0.903	0.382
MHKS	−0.118	0.203	0.086
MHPK	0.367	0.656	0.277
Set 2—Positive Mental Health			
Personal Satisfaction	0.104	0.260	0.110
Prosocial Attitude	0.503	0.882	0.373
Self-Control	−0.298	0.266	0.113
Autonomy	−0.225	0.019	0.008
Problem-Solving and Personal Achievement	0.549	0.772	0.327
Interpersonal Relationship Skills	0.284	0.666	0.281

**Table 5:** Hierarchical multiple regression analysis of factors associated with help-seeking intention among nursing students (N = 388).

Model		b	SE	β	*t*	*p*
1	(Constant)	15.814	0.290		54.467	<0.001
	Familiarity (1 = familiarity)	−0.663	0.409	−0.082	−1.621	0.106
	Year (0 = second year; 1 = fourth year)	0.988	0.375	0.133	2.632	0.009
2	(Constant)	4.197	2.347		1.788	0.075
	Familiarity (1 = familiarity)	−0.645	0.396	−0.079	−1.629	0.104
	Year (0 = second year; 1 = fourth year)	0.734	0.367	0.099	2.003	0.046
	Ability to recognise mental health problems	0.033	0.058	0.032	0.566	0.571
	Knowledge of how to seek information	0.199	0.078	0.132	2.560	0.011
	Knowledge of causes and risk factors	0.373	0.199	0.095	1.879	0.061
	Knowledge of self-help strategies	−0.177	0.154	−0.058	−1.151	0.250
	Knowledge of available professional help	0.075	0.134	0.030	0.564	0.573
	Attitudes that promote recognition and help-seeking	0.098	0.025	0.212	3.884	<0.001
	Mental health knowledge (MHKS)	0.000	0.034	0.000	0.003	0.998
	Mental health–promoting knowledge (MHPK-10)	0.005	0.031	0.009	0.166	0.868
3	(Constant)	0.789	2.890		0.273	0.785
	Familiarity (1 = familiarity)	−0.248	0.417	−0.031	−0.595	0.552
	Year (0 = second year; 1 = fourth year)	0.783	0.366	0.105	2.139	0.033
	Ability to recognise mental health problems	0.053	0.058	0.052	0.924	0.356
	Knowledge of how to seek information	0.137	0.078	0.091	1.744	0.082
	Knowledge of causes and risk factors	0.413	0.196	0.105	2.106	0.036
	Knowledge of self-help strategies	−0.165	0.152	−0.054	−1.089	0.277
	Knowledge of available professional help	0.022	0.132	0.009	0.164	0.870
	Attitudes that promote recognition and help-seeking	0.076	0.026	0.165	2.904	0.004
	Mental health knowledge (MHKS)	0.006	0.033	0.009	0.172	0.864
	Mental health–promoting knowledge (MHPK-10)	−0.006	0.032	−0.010	−0.191	0.849
	Personal satisfaction	0.075	0.065	0.074	1.156	0.248
	Prosocial attitude	−0.148	0.133	−0.075	−1.114	0.266
	Self-control	−0.114	0.085	−0.089	−1.344	0.180
	Autonomy	0.017	0.081	0.013	0.210	0.833
	Problem-solving and personal achievement	0.147	0.060	0.184	2.453	0.015
	Interpersonal relationship skills	0.150	0.075	0.121	2.000	0.046
	Model 1: R = 0.154; R^2^ = 0.024; Adjusted R^2^ = 0.019; ΔR^2^ = 0.024; ΔF(2, 385) = 4.69, *p* = 0.010 Model 2: R = 0.346; R^2^ = 0.120; Adjusted R^2^ = 0.096; ΔR^2^ = 0.096; ΔF(8, 377) = 5.14, *p* < 0.001 Model 3: R = 0.403; R^2^ = 0.162; Adjusted R^2^ = 0.126; ΔR^2^ = 0.043; ΔF(6, 371) = 3.15, *p* = 0.005; F(16, 371) = 4.50, *p* < 0.001; Durbin–Watson = 1.94; VIF = 1.07–2.49					

b = Unstandardized regression coefficient; SE = Standard error; β = Standardized regression coefficient.

## Data Availability

The data supporting the findings of this study are available from the corresponding author upon request.

## Public Involvement Statement

Members of the public were not involved in this research.

## Guidelines and Standards Statement

This quantitative study was reported in accordance with the STROBE checklist for cross-sectional studies.

## Abbreviations

The following abbreviations are used in this manuscript:

HSI	Help-seeking intention
MHL	mental health literacy
MHLS	Mental Health Literacy Scale
MHKS	Mental Health Knowledge
MHPK-10	Mental Health Promoting Knowledge Scale
MHSIS	Mental Help Seeking Intention Scale
PMH	positive mental health
+MHQ	Positive Mental Health Questionnaire
SWB	subjective well-being (SWB)
